# Constraining OCT with Knowledge of Device Design Enables High Accuracy Hemodynamic Assessment of Endovascular Implants

**DOI:** 10.1371/journal.pone.0149178

**Published:** 2016-02-23

**Authors:** Caroline C. O’Brien, Kumaran Kolandaivelu, Jonathan Brown, Augusto C. Lopes, Mie Kunio, Vijaya B. Kolachalama, Elazer R. Edelman

**Affiliations:** 1 Institute of Medical Engineering and Science, Massachusetts Institute of Technology, Cambridge, MA, United States of America; 2 Cardiovascular Division, Brigham and Women’s Hospital, Harvard Medical School, Boston, MA, United States of America; 3 Charles Stark Draper Laboratory, 555 Technology Square, Cambridge, MA, United States of America; Technion—Israel Institute of Technology, ISRAEL

## Abstract

**Background:**

Stacking cross-sectional intravascular images permits three-dimensional rendering of endovascular implants, yet introduces between-frame uncertainties that limit characterization of device placement and the hemodynamic microenvironment. In a porcine coronary stent model, we demonstrate enhanced OCT reconstruction with preservation of between-frame features through fusion with angiography and *a priori* knowledge of stent design.

**Methods and Results:**

Strut positions were extracted from sequential OCT frames. Reconstruction with standard interpolation generated discontinuous stent structures. By computationally constraining interpolation to known stent skeletons fitted to 3D ‘clouds’ of OCT-Angio-derived struts, implant anatomy was resolved, accurately rendering features from implant diameter and curvature (n = 1 vessels, r^2^ = 0.91, 0.90, respectively) to individual strut-wall configurations (average displacement error ~15 μm). This framework facilitated hemodynamic simulation (n = 1 vessel), showing the critical importance of accurate anatomic rendering in characterizing both quantitative and basic qualitative flow patterns. Discontinuities with standard approaches systematically introduced noise and bias, poorly capturing regional flow effects. In contrast, the enhanced method preserved multi-scale (local strut to regional stent) flow interactions, demonstrating the impact of regional contexts in defining the hemodynamic consequence of local deployment errors.

**Conclusion:**

Fusion of planar angiography and knowledge of device design permits enhanced OCT image analysis of *in situ* tissue-device interactions. Given emerging interests in simulation-derived hemodynamic assessment as surrogate measures of biological risk, such fused modalities offer a new window into patient-specific implant environments.

## Introduction

Simulations can enhance vascular imaging by fusing anatomy with meta-data describing physical forces [1–5]. The diagnostic potential of such integration has been suggested by an increasing number of examples, such as coronary computed tomography (CT) to derive fractional flow reserve (FFR; FFR^CT^)[6] and intravascular ultrasound (IVUS) to predict plaque progression with low shear stress [3]. As biomechanics are among the factors that influence vascular responses and clinical outcomes, there is keen interest in understanding their value as surrogate metrics of clinical risk. Yet performing imaging adequate for high-resolution computational simulation in the context of devices such as stents or scaffolds poses unique challenges.

**Fig 1:**
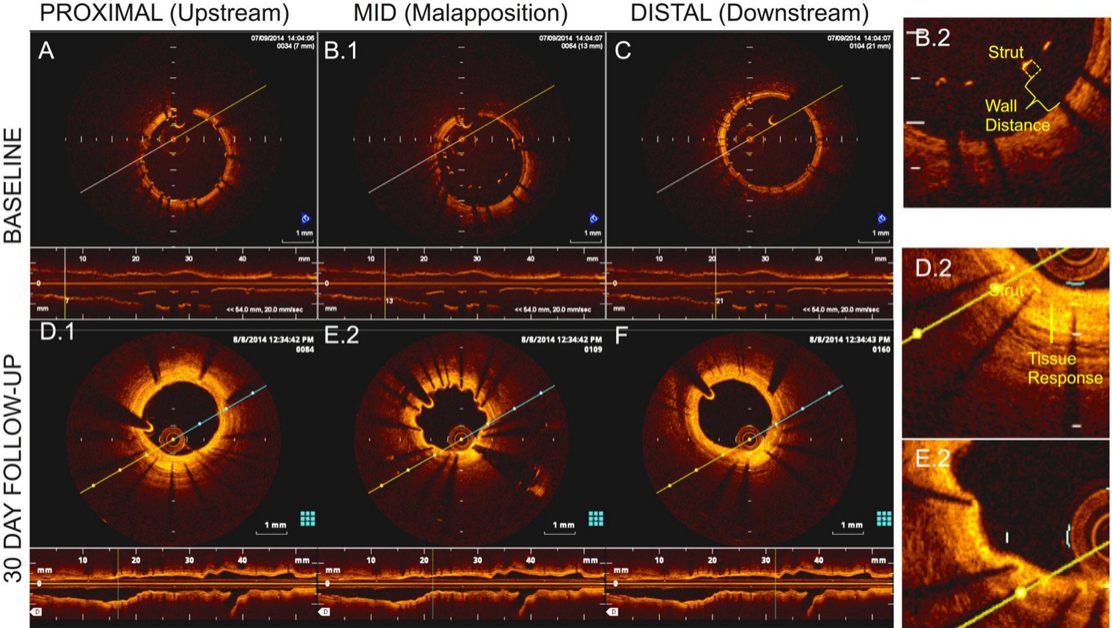
OCT demonstrating cross-sections and lumen-centered reconstructions on day 0 in (A) proximal; (B) mid; and (C) distal stent locations as well as 30-day (D) proximal; (E) mid; and (F) distal locations. The mid-section depicts strut-wall malapposition (B.2), thinly covered by day 30 (E.2). Thicker 30-day coverage was noted on apposed struts (D.2).

Optical coherence tomography (OCT) is the preferred method for resolving endovascular stent geometry *in situ* [7]. It permits visualization of device deployment at the level of individual struts (Fig 1A–1C), including imprecisions, as exemplified by strut-wall malapposition, as well as quantification of biological responses that demonstrate marked variability both locally in the vicinity of individual struts, as well as regionally over the length of the entire stent (Fig 1D–1F). Understanding the mechanisms (or hidden variables) that drive such variability is critical to further our understanding of biological mechanism, procedural risk, and prediction of clinical outcomes–all the more important given the rapid development and clinical testing of newer scaffold technologies whose properties and potential risks have yet to be fully appreciated [8]. Despite high in-plane ‘axial’ resolution (~10 μm) on OCT, out-of-plane lateral resolution remains limited (>100 μm) [7]. Regional features such as vessel curvature are poorly reproduced, as is inter-strut connectivity that is lost [9] given the space between frames. While the former issues are mostly addressable though fusion of OCT with secondary modalities like angiography that allow relative frame positioning in 3D space [10, 11], limitations regarding under-sampling remain poorly addressed. This along with implant-related artifacts that obscure local images significantly limits our ability to define device configuration *in situ* or perform simulations that rely on structural continuity [12].

We hypothesized *a priori* information on device design could minimize uncertainty between OCT frames and together with angiography, improve accuracy of OCT reconstruction and in doing so, improve description of continuous fields as exemplified with flow simulation. In a porcine coronary model of malapposed stent deployment, we demonstrate enhanced rendering of complex implant geometries relative to standard interpolation. Between-frame connectivity preserved important aspects of device-tissue architecture, permitting detailed *in situ* flow characterization both at the level of individual struts, as well as the integrated, regional level of the stent. Such image analysis frameworks that preserve across and between-frame connectivity can help refine our description and understanding of the microenvironment following device implantation.

## Methods

See Supplemental Methods (S1 File) for further details.

### Animal Studies and OCT Image Acquisition

Animal procedures were performed at an AAALAC accredited facility (CBSET Inc, Lexington, MA) in accordance with USDA Welfare Act and amendments, and the Guide for Care and Use of Laboratory Animals guidelines [13]. The protocol was approved by the Committee on Animal Care at Massachusetts Institute of Technology (Protocol Number: I00129). Yorkshire swine (n = 4, 40–50 Kg) were anesthetized by injection of Telazol (4–6 mg/kg IM) as a pre-anesthetic and then isoflurane anesthesia was delivered in 100% oxygen to facilitate endotracheal intubation. Following 6F carotid arterial access, heparin (50–200 U/kg) was administered. Coronary arteries were cannulated and iso-centered angiographic images obtained. Pain was managed during anesthetic procedure with Buprenorphine (0.01–0.03 mg/kg, IM).

Bare metal stents (BMS; 3.0 x 17 mm) of two designs were implanted–small-cell and large-cell in appropriately sized coronary arteries. Controlled under-expansion modeled suboptimal deployment. Briefly, 3.0 x 18 mm balloon catheters were modified with restrictive PET tubing (2.8 x 5 mm) over their mid portion, inducing a constrained hour-glass shape upon inflation (12 atm). Sleeves were secured using UV-cured biocompatible adhesive and catheters sterilized with ethylene oxide. Stents were crimped to the modified balloons prior to deployment (Machine Solutions, Inc., AZ).

Following deployment, frequency-domain OCT images were acquired in raw 16-bit format (C7-XR OCT Intravascular Imaging System; St. Jude Medical; Fig 1; S1 File). After intracoronary administration of 200 μg nitroglycerin, the OCT catheter (C7 Dragonfly, St Jude Medical) was advanced ~5 mm distal to the stent. OCT acquisition was performed during power contrast flushing (4–6 mL/s), with pullback of 20 mm/s for 200 μm lateral resolution or 10 mm/s for comparative 100 μm resolution. Upon blood clearance, acquisition initiated automatically (repeated for motion artifacts or inadequate flushing). After z-offset calibration, images were stored. Isocenter identification and EKG-gated angiograms (30 frames/s; paired non-orthogonal planar cine) were stored in all cases. Pre-procedurally the animals were maintained on normal pig chow diet and daily aspirin (650 mg). The day prior to survival interventions clopidogrel (300 mg) was administered.

Three animals were survived after stent implantation, for concurrent longitudinal study, and incision site was repaired. The animals survived were treated daily thereafter until euthanized on dual antiplatelet therapy (aspirin 81 mg; and clopidogrel 75 mg) and demonstrative 30-day images (Fig 1) were obtained to define biological responses. After prenecropsy angiography, each animal will be euthanized under anesthesia as per Table 1 and in accordance with accepted AVMA guidelines[14].

**Table 1:** Anticoagulant and Euthanasia Medications.

Drug Name	Dose	Route
Euthasol (Sodium Pentobarbital)	1 mL/4.5 kg or 120 mg/kg	IV
Potassium Chloride (KCl) administration under a surgical plane of anesthesia.	2 mEq/kg	IV

For acute evaluation, one animal was euthanized at the end of procedure and heart was perfused with Ringer’s lactate then perfusion-fixed (10% neutral buffered formalin). Stented segment was excised and imaged using micro-computed tomography (microCT; GE Healthcare, Milwaukee, WI) with a voxel size of 100 μm (energy: 70 kV, integration time: 16 ms), providing an *ex vivo* comparative standard.

### Image Processing and Reconstruction

#### OCT Frame Analysis

By analyzing frames in polar raw-image data format, stent struts and lumen border in each frame were automatically identified based on earlier methods [15, 16] and is described in greater detail in the Supplement (S1 File). Briefly, unique A-line signal characteristics generated by either stent or vessel wall attenuation were used as input features in a decision tree classifier that designated each A-line signal as belonging to strut, vessel wall or “other” (e.g. residual blood; 10-fold cross-validation rate of 99.8%, 99.2%, and 97.8% for successfully predicting strut, vessel wall, and “other” structures, respectively; see Supplemental Methods S1 File; Figure A in S1 File). After classifying each A-line signal, we use a ramp edge detection method [17] to locate the leading strut surface position and lumen border. After transforming to Cartesian coordinates and clustering contiguous strut surface pixels, a single pixel defining adluminal strut surface position was defined at each cluster’s centroid [18]. The true centroid of the 2D strut was obtained by projecting this strut surface position half a strut width in the direction of the vessel wall. Lumen pixel candidates were interpolated and smoothed to generate a closed lumen border.

Strut and vessel-lumen border identification was validated against those manually obtained from the console, with the average difference between lumen and stent areas between the two methods calculated as 0.42 ± 0.13 mm^2^ (n = 4 vessels, 5.4% ± 0.19%, n = 62 frames, r = 0.97) and 0.2 ± 0.17mm^2^ (3.08% ± 0.36%, n = 57 frames, r = 0.98), respectively (S1 File).

#### Enhanced reconstruction

The enhanced method leveraged OCT fusion with planar angiography (2+ images) and prior stent design knowledge. From original angiography, binary images were generated by thresholding. Arterial edge detection and skeletonization defined arterial centerline projections on each cine frame. By defining image points fitting epipolar constraints on two images, the projection matrix relating 3D points to 2D projections was determined (S1 File). By defining the 3D vessel centerline and angiographic landmarks, sequential OCT frames were then mapped into 3D space, using a method described previously for IVUS and angiographic fusion[19] with minor modifications made for OCT implementation. Briefly, a subset of images was selected that included a stented segment and proximal or distal side branch. The first frame position (either upstream or downstream of the stent depending on side branch position) was determined by identifying the frame furthest from the stent where the side branch centerline was one radius away from the stented branch centroid. Absolute first frame orientation was defined by rotating the OCT frame until the side branch aligned with the side branch centerline defined on planar angiography. Each subsequent frame was then placed perpendicular to the centerline, and rotated an amount defined by a standard sequential triangulation algorithm[19]. Frames were spaced so that distance between the catheters location on successive frames was 0.2mm (corresponding to equivalent pullback speed).

While imaged content on stent geometry was not available in the 0.2 mm gaps between frames, information on stent design was used to constrain stent configuration.This was done by generating idealized stent skeletons from NURBS curves constrained by control points obtained from the known stent designs. This skeleton was computationally ‘deployed’ by registering idealized control points to strut centroids in the transformed OCT frames (defined in the OCT frame analysis) using a non-rigid point matching algorithm (Figure C in S1 File) [20]. Over the deformed skeleton, known strut cross sections were swept (Figure C in S1 File) [21]. A Boolean operation united surfaces into the final stent geometry (watertight solid), required for simulation. Relative fit of deformed stent geometries were calculated by measuring displacement error of strut landmarks between OCT and reconstruction (Figure D in S1 File). Lumen border contours in transformed OCT frames were lofted over [21] to generate continuous surfaces. All algorithms for OCT frame analysis and reconstruction were implemented in Matlab (MathWorks, Inc.).

#### Standard reconstruction

**Fig 2:**
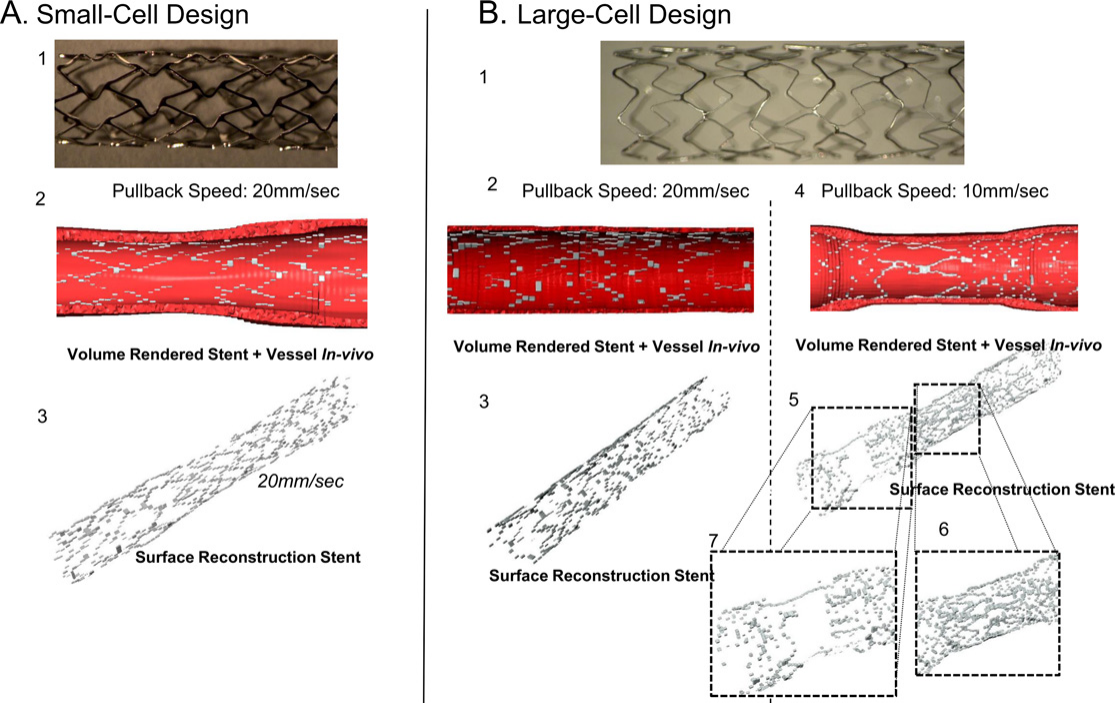
Reconstruction of (A) small-cell; and B. large-cell stent designs using standard segmentation/interpolation. Frequency-Domain OCT provides estimates of stent strut and lumen position on each frame, which can be visualized as a volume rendered image for (A.2). small-cell; and (B.2, B.4) large-cell designs at fast (B.2); and slow (B.4) pullback speeds. Despite lumen-centering, reconstruction yielded stent geometries with significant discontinuities (A.3, B.3) with little change despite slower pull back (doubled resolution; (B.4, B.5)). Regions where stent diameter varies significantly are most poor reproduced (B.6, B.7).

Comparative geometries were reconstructed using standard segmentation and interpolation schemes for 3D iso-surface meshes. Strut and lumen contours were defined from OCT frames analysis. Strut cross-sections (100 μm x 100 μm small-cell; 80 μm x 80 μm large-cell) were built around detected struts. Frames were re-centered about lumen centroids, and stacked at 0.2 mm spacing (0.1 mm for slower pullback). As these algorithms require image stacking along a straight axis, a straight lumen centerline was enforced. Voxels representing stent and lumen volumes were created by laterally extruding segmented contours on each slice a distance half the inter-slice thickness, yielding volume rendered images of stent and vessel wall (Fig 2A.2). Surfaces were built around voxels using an enhanced marching cubes algorithm (SCAN-IP) [22].

### Simulations

Simulation of steady laminar flow fields in and around a single implanted malapposed device derived from both standard interpolation and enhanced methods was based on our experience modelling in idealized stent strut [23–26]Blood was modeled as a non-Newtonian fluid. Boundary conditions included fully developed inlet flow (mean velocity 0.25 m/s), no slip walls and zero outlet pressure. Governing equations were solved numerically (ANSYS Fluent v15, Canonsburg, PA). A second-order upwind scheme was used for momentum variables and pressure-velocity coupling with the SIMPLE algorithm. Convergence was defined when solution residuals were below 10^−8^. Mesh independent solutions were confirmed when <3% difference in probe point values occurred between meshes (final mesh size of 4 x 10^8^ volume elements).

Discrete measurements of device configuration and flow parameters were obtained from the 3D continuum by sampling the domain at 20 equidistant planes perpendicular to the vessel centerline. Rays were constructed on each plane, extending from vessel centerline to each “strut point,” defined as the intersection of the stent skeleton and respective plane. The intersection of a particular ray and wall surface mesh[27] represented “wall position” of a given strut point (See Figure E in S1 File for schematic). Wall distance (WD) was reported as the distance from strut point and corresponding wall position after accounting for stent thickness, consistent with standard OCT definitions. We assumed struts had uniform thickness of 80 μm with 20 μm cushion for OCT blooming artefacts [7]. Therefore struts with WD>100 μm were defined as malapposed; WD>0 μm and <100 μm were partially embedded. Wall shear stress (WSS) was assessed at each wall position. When uncovered struts contacted the wall, WSS was defined on the adluminal strut surface, though zero at true vessel wall. Strut-associated shear rate was defined as the maximum shear rate in a 160 μm radius around a given strut.

Qualitatively comparable 2D flow simulations around single strut elements (80 μm x 80 μm) at varying degrees of wall displacement (80, 160, or 320 μm) were performed, as were simulations with six sequential struts arranged with 1 mm spacing described elsewhere.[23]

### Statistical Analysis

Correlations between variables of interest were assessed using the Spearman’s correlation coefficient. Comparisons between correlations were made using analysis of covariance (ANCOVA). Continuous variables (Wall Distance -“WD”- and lateral position along implant length) were also compared after binning into 3 levels (zones 1, 2, and 3 for wall distance, signifying < 100; 100–200; and > 200 wall displacements, respectively; and proximal, mid, and distal for the first, middle, and last third along the stent length). Bias was loosely identified as the ratio of mean values in each bin between enhanced and standard methods; noise as the ratio of standard deviations. Univariate or multiple linear regression was performed to quantify amount of flow variance explained by continuous strut position variables (WD and/or position along stent length). For multivariate hypothesis testing in samples of OCT and reconstructed landmarks, Hotelling’s T^2^ statistic was used. p-values < 0.05 were assumed statistically significant.

## Results

### Enhanced OCT Analysis

Stent designs with controlled under-expansion (Fig 2A and 2B) were imaged and geometries reconstructed. As expected, standard interpolation introduced significant discontinuities (Fig 2A.3, 2B.3 and 2B.5). Reconstruction volume fraction was defined as summed volume of all rendered stent segments divided by the experimentally derived stent volume (average of 10 mass/density measurements). Small- and large-cell stents were poorly reconstructed using the standard technique (volume fractions of 13.5% and 38.2%, respectively). At double lateral resolution (10 mm/s vs. 20 mm/s pullback), volume fraction failed to improve (29.2%; Fig 2B.2), reflecting the many factors contributing to interpolation beyond lateral resolution alone. We observed segments over which stent diameters changed significantly were most poorly reproduced with standard interpolation (Fig 2B.6 and 2B.7).

**Fig 3:**
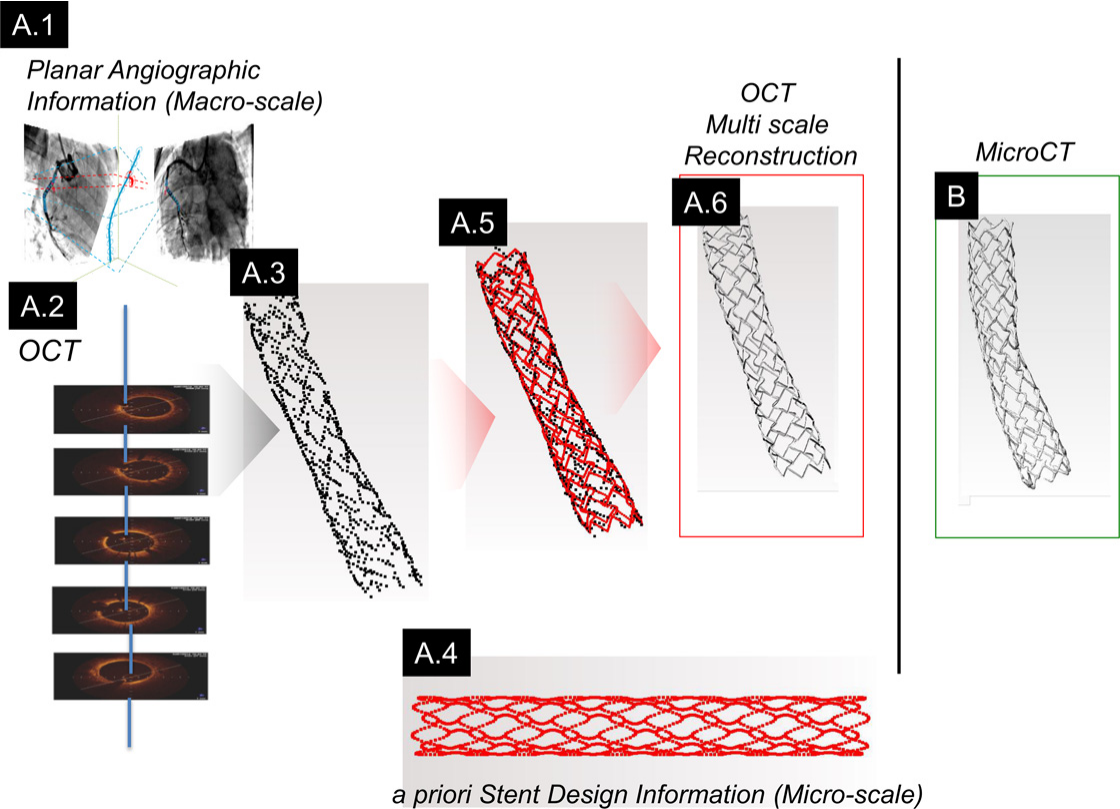
Schematic workflow of the enhanced method. (A.1) Paired planar angiography images and (A.2) OCT are fused to recover 3D spatial features from which strut positions are identified (A.3). (A.4) Prior knowledge of stent design embodied as a point cloud of spatial information is fit to the OCT/Angio-derived strut positions (A.5). (A.6) A final stent geometry is recovered by extruding known strut cross-sections along the path defined by the morphed point cloud. (B) These data visually matched *ex vivo* microCT images.

**Fig 4:**
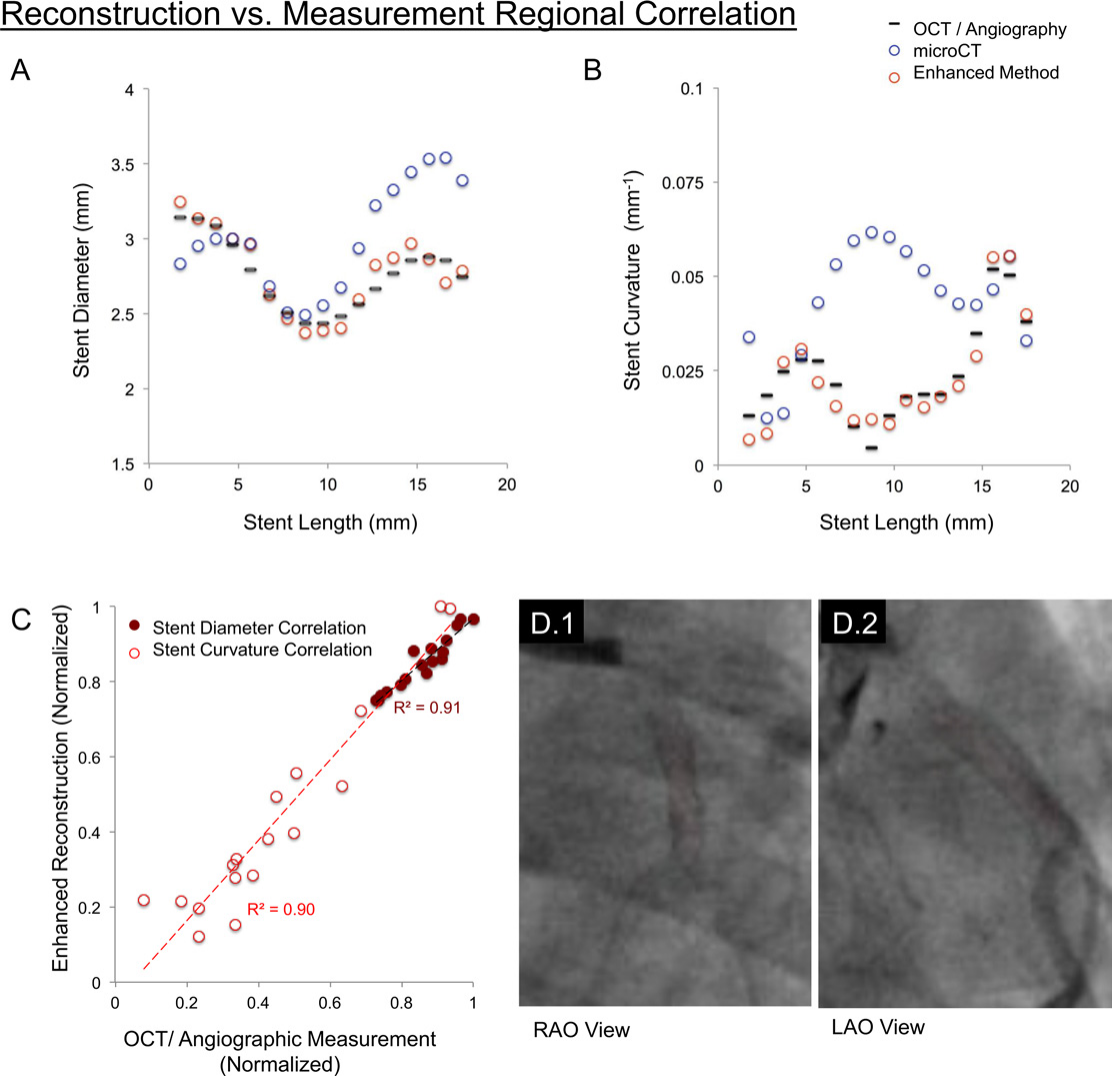
Discrete sampling of continuous enhanced reconstruction geometries facilitated comparison with OCT measurements. Enhanced reconstruction (red circles) aligned closely with OCT/angiographic measurements (black lines), demonstrated for both (A) stent diameter; and (B) curvature. (C) Quantitatively, these shape parameters correlated strongly (r^2^ = 0.91 and 0.90, respectively) demonstrating recovery of implant shape at both local and regional scales. In contrast, mid-stent curvature on microCT measurements diverged (blue circles; in Fig 4B). (D.1,2). Planar angiography views with the centerline (red) superimposed through the stent demonstrate the relative straightness of the stent mid-region, suggesting microCT divergence was attributable to deformations of vessel geometry introduced upon explantation and processing.

OCT fusion with angiography and stent design information improved 3D reconstruction (Figs 3A and 1A.6). Reconstruction volume fraction increased for small- and large-cell stent designs (85.6% and 85.4%, respectively). Similar to *ex vivo* microCT, the enhanced method generated continuous structures (Fig 3A.6 and 3B). Similar stent cross-sections were observed between OCT-angiography, microCT and enhanced reconstruction. Regional parameters (diameter, curvature; Fig 4C) and local shape matched closely between OCT measurements and corresponding enhanced reconstruction landmarks (average strut displacement error 16.6 μm; p = 0.67; Figure E in S1 File) [28]. In contrast, curvature diverged with microCT, stressing the impact explantation can have on distorting histological geometries (Fig 4A–4C).

### Local Flow Over Struts

**Fig 5:**
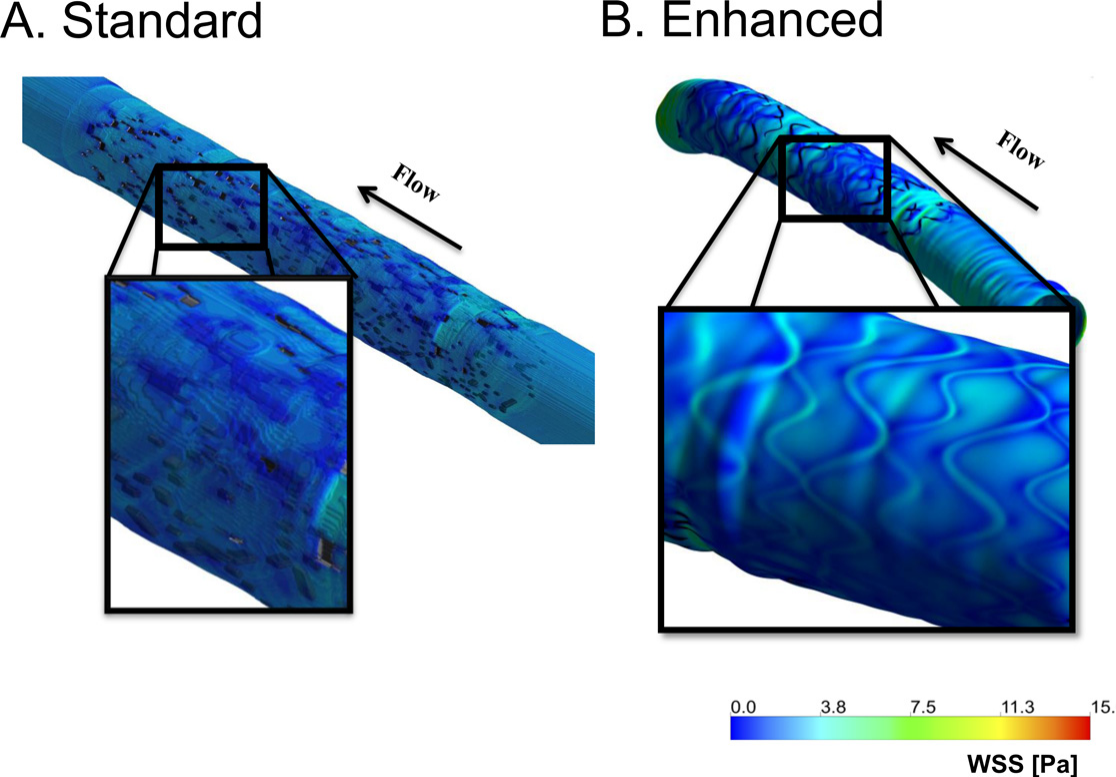
Wall shear stress (WSS) in a geometry defined using (A) standard; or (B) enhanced reconstruction. The standard approach yielded local discontinuities on WSS contour plots. In contrast, WSS is highly resolved using the enhanced method.

**Fig 6:**
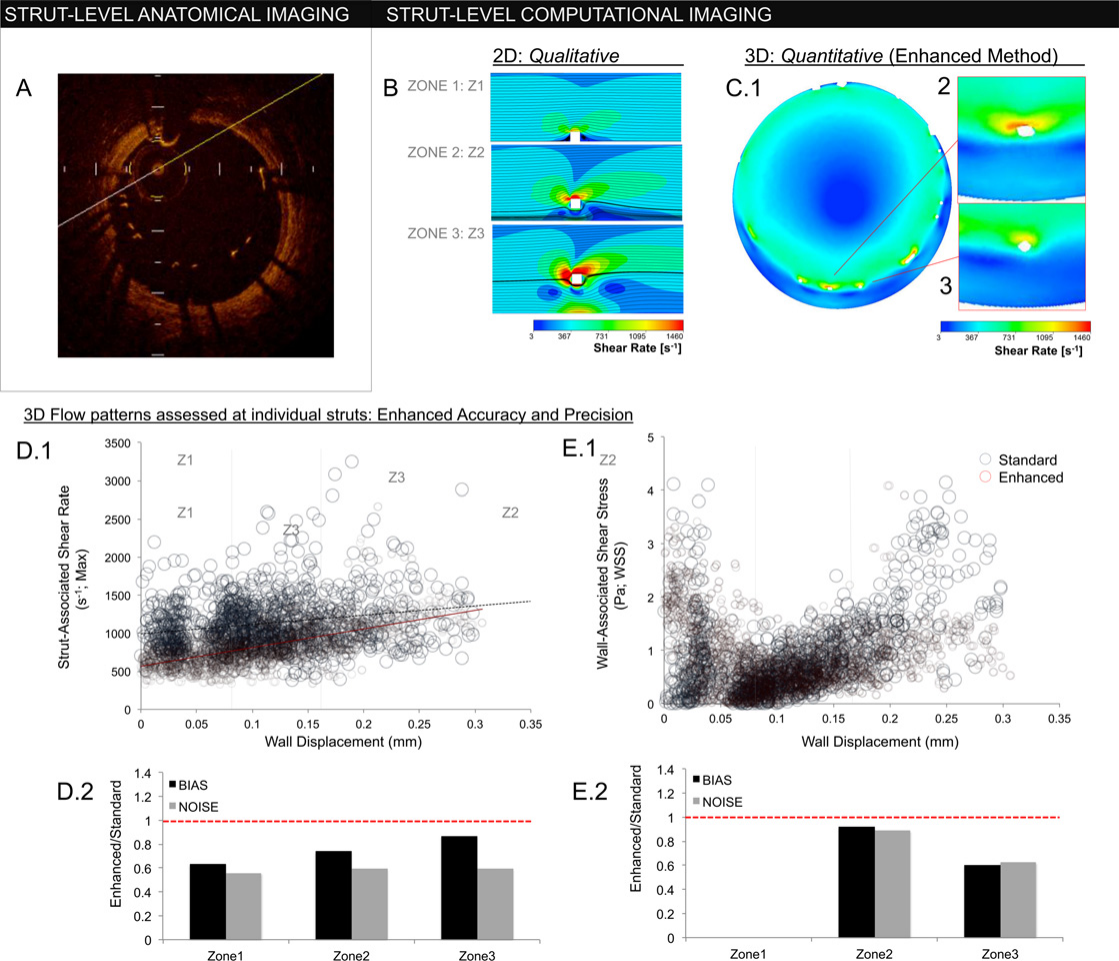
(A) In-plane OCT frame depicting strut-wall displacement (WD). Struts with WD greater than a single strut thickness (>80 um) are malapposed. (B) 2D simulations of flow around an idealized ‘strut’ (white square; 80 μm x 80 μm). Flow disturbances are influenced by WD, demonstrated for WDs of 80 μm, 160 μm, and 320 μm in zones 1, 2, and 3, respectively (zone 1≤80 μm; 80 μm <zone 2≤160 μm; 160 μm<zone 3). Enhanced reconstruction permits detailed hemodynamic characterization in 3D patient-specific settings. (C.1) In-plane, cross-sections of the implant hemodynamic microenvironment. As with 2D simulations, flow disturbances around struts (white) increase with WD. (C.2), (C.3) Unlike idealized 2D simulations, inter-strut variability exists despite similar anatomic strut position–a phenomenon reliant on 3D effects. Hemodynamic accuracy requires accurate definition of implant geometry, demonstrated by comparing flows generated from the enhanced and standard interpolation methods. (D.1) Strut-associated shear rate correlated strongly with WD in the enhanced method (red circles; slope = 2447 μm^-1^s^-1^; r^2^ = 0.45), though the association was blunted and dispersed with standard interpolation (blue circles; slope = 1221 μm^-1^s^-1^; r^2^ = 0.05). (D.2) Bias, defined as ratio of mean value in each zone between the enhanced and standard method, was reduced in all zones. Noise, defined as ratio of standard deviations in each zone, was also reduced. (E.1), (E.2) Wall shear stress (WSS) demonstrated a “U” type relationship, being high in zone 1 when struts contacted vessel wall (here, WSS is defined on the upper surface of the strut). As contact was lost, WSS decreased reflecting flow shielding between strut and wall (zone 2). With further strut displacment (zone 3), WSS recovered to levels observed in zone 1. Again, the enhanced method (red circles; in E.1) demonstrated reduced noise and bias compared to the standard approach (blue circles; E.1). The largest discrepancies in the standard method were in regions of highest malapposition (zone 3), where discontinuous reconstructions cannot maintain an adequate flow barrier.

We next compared flow simulations using each reconstruction method. The hemodynamic microenvironment was far more resolved with the enhanced scheme (Fig 5). We quantified differences at the in-plane, cross-sectional level as well as the inter-frame, regional level (proximal to distal stent). Cross-sectional strut apposition was characterized by strut-wall displacement, WD, akin to standard OCT definitions (Fig 6A).

We and others have shown that thrombotic [23] and hyperplastic responses [29] to WD are not simple, monotonically increasing functions that track with extent, but rather have non-linear relationships with intermediate degrees of malapposition potentially tempering responses. Idealized models have been invaluable in helping explore parameters that may better explain such observed responses. Akin to prior findings, comparative 2D simulations demonstrate increasing strut-associated shear rates with increasing WD [29], while wall-associated shear stress first decreased, then increased with strut displacement into the free stream (Fig 6B) [23].

Enhanced simulations verified these qualitative 2D relationships are retained in 3D implant microenvironments *(*Fig 6C.1). Strut-associated maximum shear rate correlated strongly with strut displacement given exposure to higher free stream velocities (red circles, Fig 6D.1; Spearman correlation coefficient 0.59, p < 0.0001). As struts shield the wall from centerline flow (Fig 6C.1), wall-associated shear stress decreased as struts lost contact with the vessel wall (WD = 80 μm; zone 1), then rose with intermediate (zone 2) and high (zone 3) WD (Fig 6D.2) as flow reconstitutes below struts.

There was reduced correlation between WD and strut-associated shear rates with standard interpolation (blue circles, Fig 6D.1; Spearman correlation coefficient 0.161, p < 0.0001). While some shear rates in zones 2 and 3 were greatly elevated, others were blunted in a variable manner driven by local and regional structural irregularities (see below). Overall, noise (shift in standard deviation) and bias (shift in magnitude) were increased with the standard approach as compared to the enhanced method, yielding divergent strut-associated shear values (ANCOVA p < 0.0001; Fig 6D.2). For WSS, largest overshoot occurred in zone 3 struts (highest malapposition; Fig 6E.2) due to loss of wall-shielding from structural discontinuities introduced with standard interpolation.

### Regional Flow through Stents

**Fig 7:**
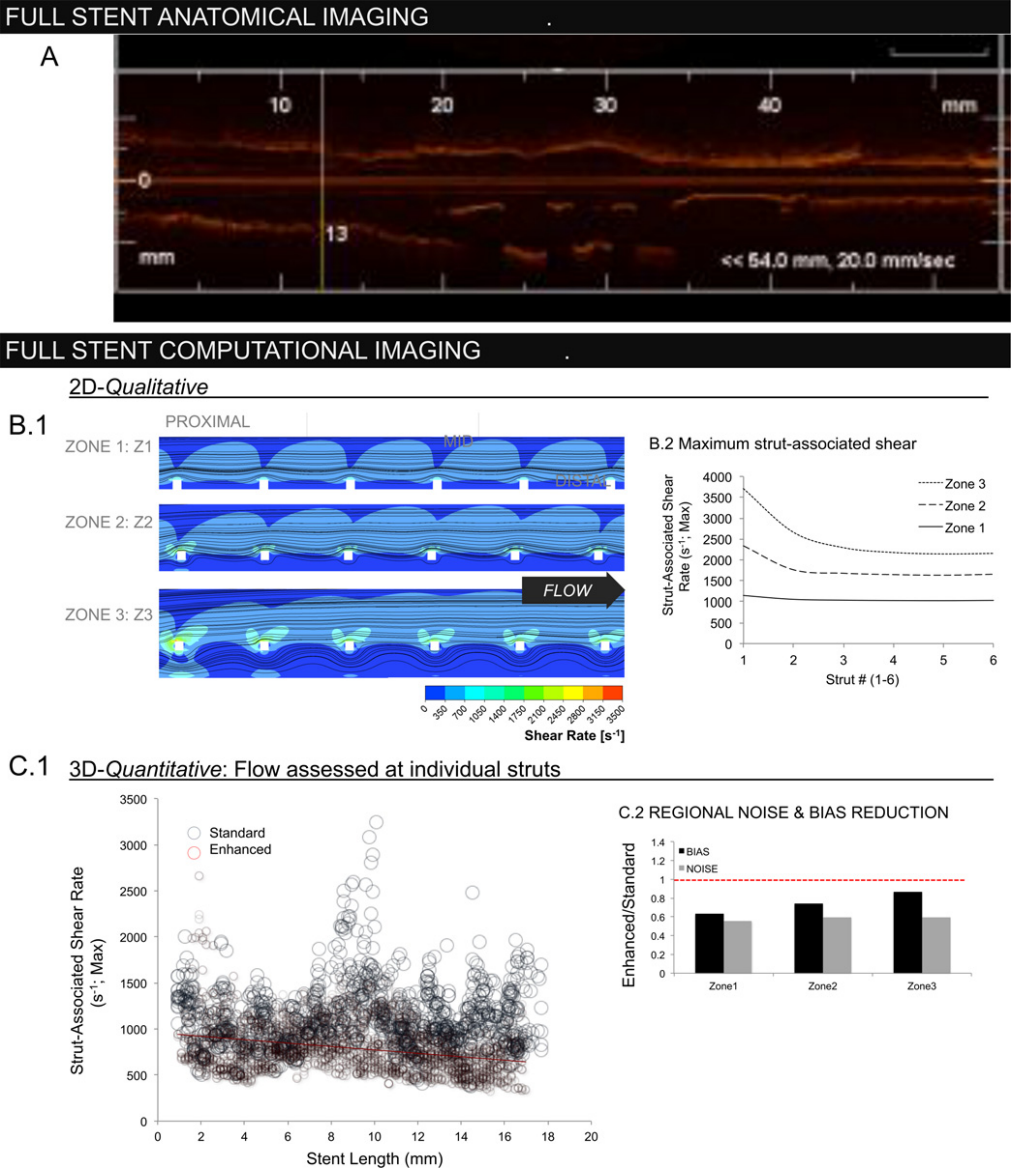
(A) Lateral OCT frame demonstrating regional variations in the stented vessel (direct output from OCT console). (B.1) 2D flow reconstructions demonstrating the qualitative impact of strut-wall displacement (WD) as a function of lateral position along the stent length. (B.2) Largest flow disruptions occur in proximal struts with highest WD (zone 3), diminishing distally as upstream struts create a flow barrier. (C) While the enhanced method (red circles) was able to recover this regional trend, as demonstrated by negative correlation between maximum strut-associated shear rate and position along the stent length (slope = -18.5mm^-1^s^-1^; r^2^ = 0.09), absent with the standard method (blue) (slope = 1.4 mm^-1^s^-1^, r^2^ = 0.0003). Loss of inter-strut interactions due to discontinuous stent geometries generated by the standard method resulted in systematic overestimation of shear rate.

We evaluated flow simulation across stent length (Fig 7). 2D simulations demonstrated that the local increase in strut-associated shear rate with WD is also a function of sequential position given downstream shielding by upstream struts (Fig 7B). Enhanced 3D reconstructions demonstrated similar reduction in strut-associated shear as a function of lateral position (Fig 7C; Spearman correlation coefficient 0.09; p < 0.002). The trend was absent with standard interpolation (Spearman correlation coefficient 0.0003; p = 0.992).

Regional bias (magnitude) and noise distributions (standard deviation) of strut-associated shear rates were reduced for the enhanced method as compared with the standard approach at proximal, mid and distal segments (Fig 7C.2). Discontinuities introduced with standard interpolation lead to loss of position information along implant length resulting in systematic flow discrepancies that were highest distally (Fig 7C, inset). As geometries produced via the standard approach were variably discontinuous, lateral shielding effects were also variable and contributed to noise (Fig 6.D.1 and 6.D.2).

**Fig 8:**
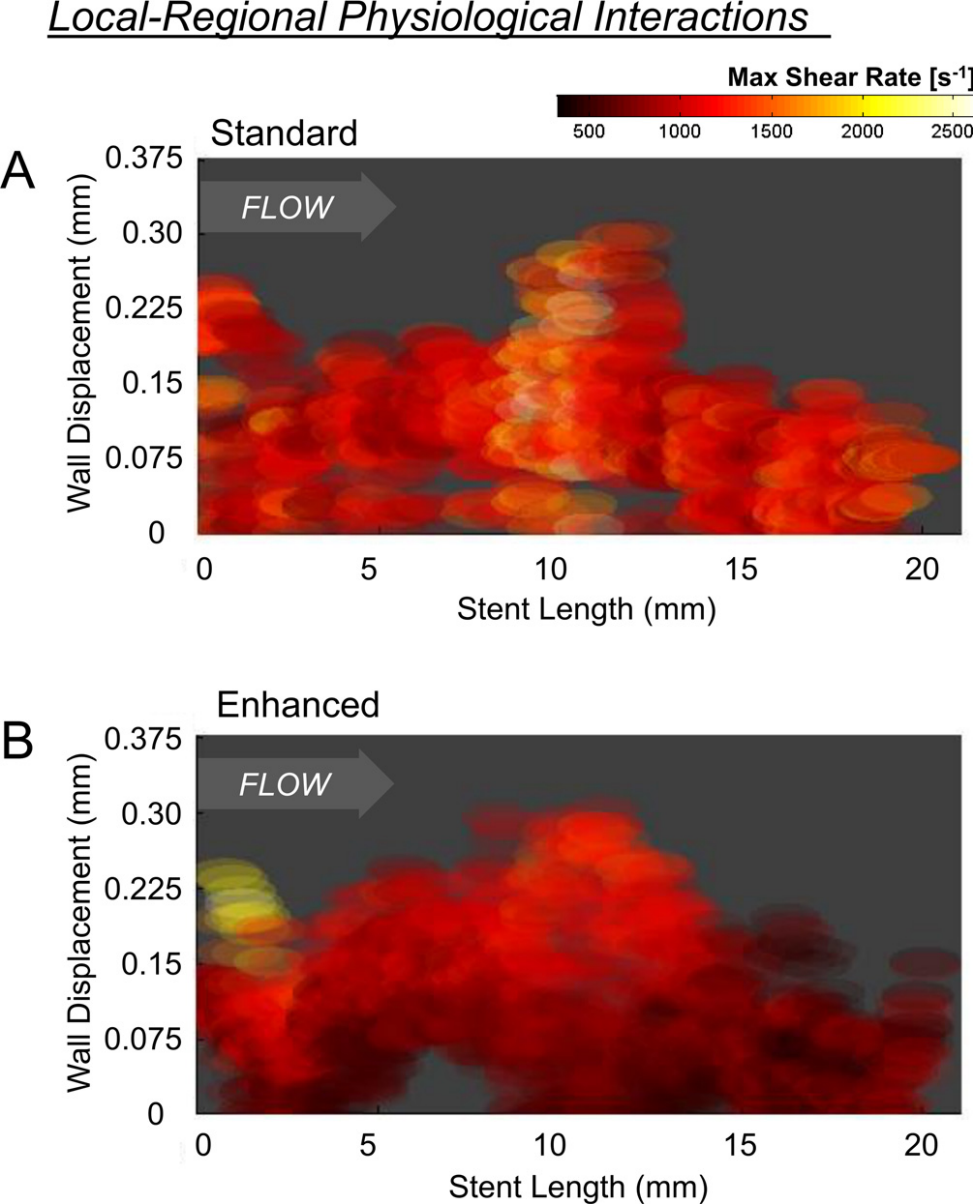
Depicted is the maximum strut-associated shear rate as a function of strut position relative to radial wall displacement (y-axis) and axial location in the stent (x-axis; from proximal to distal). (A) Stent-level organization in shear rate was largely absent in the standard method, as demonstrated by the relatively homogenized nature of shear stress patterns across the stent. (B) This is in contrast to flow simulation performed using the enhance reconstruction method, were organized and more graded flow patterns were observed at both local (WD; y-axis) and regional (stent-length; x-axis) levels as shear stress smoothly transitioned from the proximal to distal end of the stent.

Important for the identification of latent variability, while the enhanced approach reduced noise, it did not suppress true inter-strut flow variation (Fig 6C.2 and 6C.3). While noise represents unorganized variability, true inter-strut differences represent organized variation arising in the context of interactions with surrounding elements. Given loss of surrounding structures, standard reconstructions poorly captured interactions between WD and lateral strut position (Fig 8A; color scale depicts maximum strut-associated shear rate); only a fraction of flow variability was explainable by WD and regional position (multiple regression r^2^ = 0.055). In contrast, regional organization in the flow environment was apparent with enhanced reconstruction. Displaced proximal struts were associated with markedly higher shear rates compared to similarly displaced mid- or distal-segment struts and the overall flow pattern smoothly transitioned from the proximal to distal end of the stent. Nearly 50% of shear rate variance was explained by strut WD and regional position (multiple regression r^2^ = 0.47; Fig 8B).

## Discussion

Defining the biomechanical consequence of endovascular device implantation *in situ* is challenging. While the variables driving patient specific responses to intervention remains only partly characterized, features must be considered across scales ranging from microns to centimeters, especially difficult in the presence of metallic implants that produce artifacts and obscure local anatomy using standard imaging. We describe a framework that enables OCT analysis of endovascular implants through fusion with angiography as well as prior knowledge of implant geometry. Incorporation of design information allowed us to interpolate device structures, as they rest *in situ*, yet which are not typically defined given gaps between imaging frames. High fidelity, continuous reconstructions enabled simulations of the device-laden hemodynamic microenvironment, accurately recovering both local and regional flow effects.

### Knowledge-Fusion to Image Implants

Frequency-Domain OCT permits anatomical assessment of procedural success and vascular responses [7, 30]. Despite axial precision (~10 μm), uncertainty exists between and across frames. Several methods have been developed to facilitate OCT (or IVUS) spatial reconstruction [3, 5, 31–33]. One approach (standard interpolation) is lumen-centric yielding artificially straight segments [2, 7]. Another shifts to a catheter-centric reference [3, 31], where catheter position is defined using a secondary modality such as planar angiography that positions frames relative to one another along the catheter trajectory. Our enhanced method incorporated catheter-centricity, thus recovering regional features such as vessel curvature.

Along with sequential frame positioning, defining implant geometry requires intra-frame reconstruction. Recently, one group demonstrated OCT reconstruction of bioresorbable vascular scaffolds using a marching cube interpolation algorithm similar to our standard approach [2]. Interpolated geometries permitted flow simulation with sufficient fidelity to correlate hemodynamic parameters and neointimal response. This study demonstrates the importance of characterizing local flow parameters, though was constrained to evaluating straight, well-apposed segments stented with thick-strutted (150 μm) devices, in contrast to thin-strutted (<100 μm) devices with potentially complex patterns of apposition. In such cases, standard interpolation recovers implant geometries poorly (**Fig 2**).

Our enhanced method interpolated between frames based on paths constrained by known stent geometries, reproducing continuous structures that conformed to OCT frame data yielding a strut-strut mean error of 16.6 μm. While there was reconstruction volume loss, this was attributed to assumptions made in idealized stent designs and the empiric way in which true stent volume was derived. The similarity in recovered fraction between stent designs (85.6% versus 85.4%) indicates methodological robustness, important for generalizable applications across devices and implantation scenarios (in contrast to the standard approach where volume fraction varied from 13.5% and 38.2%). There is potential for such knowledge-fused reconstructions, in various applications, such as the characterization of struts as they jail bifurcating vessels or the variable patterns that arise from overlapping devices.

### Implant Hemodynamics

Powerful computational tools are now opening the door to enable simulation as an alternative means of deriving diagnostic biomechanical markers. Through simulation, hemodynamic properties can be resolved at high resolution and at decreased invasiveness as exemplified by FFR^CT^ [6]. While this technique makes use of coronary CT to identify fractional flow gradients at mm scales relevant to stenoses, it is unable to define wall anatomy with sufficient resolution to characterize the near-wall hemodynamics that directly impinge on local biology [3, 31]. Clinical significance of near-wall flows was demonstrated in the PREDICTION trial, where IVUS-derived patient-specific flows in non-stented regions predicted plaque progression and follow-up intervention [3]. In contrast to non-stented geometries, near-wall flows following stent/scaffold implantation are distorted significantly. Numerous models demonstrate the impact stent design[23, 34, 35] and deployment [23, 36] have on altering flow, yet extending this hemodynamic understanding *in vivo* requires techniques that precisely define *in situ* implant and vascular wall features.

We simulated *in situ* implant physiology and defined impact on near-wall flows. Qualitatively, our enhanced method identified patterns observed in 2D models created here (Figs 6B, 6D, 6E; 7B and 7C) and based on those created previously [29]. Quantitatively, our enhanced approach reduced bias and noise as compared to standard approaches in which even basic hemodynamic trends such as downstream shielding by upstream structures were obscured (Fig 7). Preservation of inter- and intra-frame structure/flow interactions was essential to characterize the hemodynamic microenvironment. While flow patterns over individual struts are variable, variability arises in the context of organized flow patterns that are influenced both by strut-wall displacement as well as sequential strut position along the length of the stent (Fig 8B). This variability is distinct from the locally and regionally unorganized patterns produced through the standard approach (Fig 8A). As biological response to device implantation demonstrates local and regional variability, it is important that diagnostic tools used to characterize procedural success similarly preserve the local and regional contexts that contribute to early and late response.

## Study Limitations

This study demonstrates geometry reconstruction and the impact of discrete versus continuous structural representations for characterizing continuous properties as exemplified through flow simulation. We have specifically not addressed uncertainties in flow boundary conditions, and have used a mean flow rate consistent with a prototypical coronary flow. Vessel specific flow rates can be measured from the contrast dilution speed in coronary angiography [37], though this will be considered in future work. This method has been used extensively for simulation in IVUS-generated native vessels [3, 38], but it has not been explicitly validated when used in OCT-generated stented segments.

Our manuscript focuses on the importance of method rather than the correlation with biological response. Ongoing studies will identify more extensively how near-wall flows impinge on local biology and the extent to which refined characterization improves prediction beyond anatomic characterization alone.

## Conclusions

Through integration of OCT, angiography and stent design knowledge, we provide a novel means of implant reconstruction that can enhance the ability to characterize implant microenvironments with regards to both geometric device position and simulation-based flow characterization. While the framework described was applied to stent evaluation, it provides a potential means to enhance *in situ* characterization of implants—stents, scaffolds and otherwise. The clinical implications of such improved characterizations require further exploration and validation.

## Supporting Information

S1 File Supplemental Methods. (DOCX)
