# Supplementary material for: Constraining OCT with Knowledge of Device Design Enables High Accuracy Hemodynamic Assessment of Endovascular Implants
Source: PLoS One. 2016 Feb 23;11(2):e0149178. doi: 10.1371/journal.pone.0149178 (PMC4764338; doi:10.1371/journal.pone.0149178)
Supplement: S1 File — (DOCX) [file pone.0149178.s001.docx]

­ Supplemental Methods

Supplement Table of Contents

[OCT Frame Analysis 2](#_Toc442511078)

[Method development 2](#_Toc442511079)

[Validation 4](#_Toc442511080)

[Enhanced reconstruction method: Vessel Centerline Reconstruction 4](#_Toc442511081)

[Method Development 4](#_Toc442511082)

[Validation 5](#_Toc442511083)

[Enhanced reconstruction method: Point Set Registration and Surface Reconstruction 5](#_Toc442511084)

[Method Development 5](#_Toc442511085)

[Validation 6](#_Toc442511086)

[Supplemental Figures 7](#_Toc442511087)

[Supplemental References 12](#_Toc442511088)

Supplemental Methods

Our supplement details pertinent methods used for geometry reconstruction from optical coherence tomography (OCT), and flow simulations. In our description of reconstruction methods, we first describe the automated method of OCT frame analysis that uses image processing to detect and classify strut and lumen-vessel wall boundaries. Second we outline our reconstruction of vessel centerline required for transformation of OCT features into 3D point clouds. Fusion of OCT frames and vessel centerline from angiography was detailed in the manuscript, and not further elaborated in the supplement. We then describe how a continuous stent structure is defined from 3D point cloud strut data. We finally describe how OCT measurements are co-registered between serial time points. When appropriate, method validation is also provided.

## OCT Frame Analysis

### Method development

Fourier-domain OCT acquires scans at multiple depths (termed axial-line scans; A-lines) from an imaging wire that rotates inside a fluid-filled polymer tube[[1](#_ENREF_1)]. With one revolution (i.e. 360°), a complete cross-sectional frame can be acquired. At a pullback speed of 20mm/sec, up to 271 of these frames along the vessel length were acquired with distance of 50mm separating each segment length-wise. While stent struts and vessel borders are visualized on such OCT frames, the proprietary software accompanying the OCT system (LightLab Imaging Inc., Westford, Massachusetts) does not allow for the automated detection that was critical for our high processing needs. We therefore developed a method that automatically detects and classifies strut position and lumen borders from OCT frames. We did this by **classification** of features from A-line scans followed by **edge detection** of the signal within the A-line.

**Classification:** A-lines were classified as belonging to strut or lumen using features extracted from the “scale-space signature” of the signal. This signature was generated by convolving the 1-D A-line signal, $f(r)$ where r is the axial distance, by a mother wavelet $\psi(r)$

$Wf(s,r)=\frac{1}{s}\int f(r)\psi\left( \frac{r-a}{s} \right)\mathrm{dx}$. (1)

Here *a* and *s* are the shift and scale parameters, respectively. The scale space signature of the A-line signal is the local maximum of the wavelet transform with the Laplacian-of-Gaussian (LoG) wavelet at each scale $s=\{0:2^{0.1}:2^{8}\}$, referred as the “LoG response”. Tsantis et al.[[2](#_ENREF_2)] have demonstrated that the LoG response of metallic struts are characteristically different to that of tissues (Supplement Figure A.B). We similarly extracted features from the LoG response (Supplemental Table 1) and used these features to train a machine learning model based on a decision tree algorithm (Bootstrap aggregation) to classify the A-line signal into three groups: (1) strut and (2) lumen and (3) “other” structures (residual blood).

Table 1: Table of Extracted Features used to classify lumen and stent strut structures in OCT images

| **Features Derived from Wavelet Transform** |
| --- |
| Scale s=s*, location of maximum Log Response |
| Maximum LoG Response max$\left\vert Wf(s,x) \right\vert$ |
| Position on radial axis of maximum LoG Response at scale s=s* |
| Standard deviation of LoG Response |
| Mean LoG Response |
| Skew of LoG Response |
| Position on radial axis of maximum DoG Response at scale s=s* |
| **Signal Features** |
| Max(signal) |
| Mean (signal) |
| **Neighbourhood Features** |
| s* of four closest neighbours |

As the guidewire is metallic and has a similar scale space signature as “struts”, a semi-automated approach was used to avoid misclassification errors. Briefly, the guidewire was selected manually on the first frame, and then a minimum spanning tree algorithm automatically identified the guidewire on each subsequent slice along the pullback length. Entities initially classified as “struts” but characterized as “guidewire” based on this algorithm were reclassified as “other”.

**Edge detection:** Once A-lines had been classified, edge locations of the strut and lumen were obtained using another wavelet transformation (akin to a Canny edge detector[[3](#_ENREF_3)]). Edges of the strut (abluminal side) and lumen/tissue interface represent a step edge. To optimally detect these edges, the A-line signal was convolved with the normalized first derivative of the Gaussian wavelet. This wavelet has a characteristic scale $s^{*}$ taken from the LoG response for either strut or lumen classification noted above. A-lines classified as “other” were not considered. Adjacent strut pixels in the polar image were clustered using connected components labeling[[4](#_ENREF_4)], and each cluster’s centroid was used to define a single pixel defining abluminal strut surface position (as designated by the red cross in Supplemental Figure A.C). . Lumen pixel candidates were interpolated and smoothed to generate a closed lumen border (as designated by the green boundary in Supplemental Figure A.C). Strut surface pixels and lumen border pixels were transformed into Cartesian coordinates. The true centroid of the 2D strut was obtained by projecting the strut surface position by half a strut width in the direction of the vessel wall.

### Validation

Accuracy of extracted strut and lumen dimensions was computed using a candidate data set in the LCX vessel of a single pig. Each A-line within the stented segment of 80 slices (≈40100 A-line signals) was classified as belonging to strut, lumen or “other” categories (including contrast or artifacts in the tissue). A classifier was trained with these categories as outputs and the extracted features of the A-line signal from the wavelet transform as the inputs. Receiver-operator characteristic curves showed an area under the curve (AUC) measurement for classification of strut candidates to be 0.99, the lumen ~1.0 and for “other” structures 0.99, with sensitivity and specificity values ranging from approximately 0.94-1.0 and 0.83-0.99, respectively (Supplemental Figure A.C). This method of feature detection had a 10-fold cross-validation rate of 99.8% in successfully predicting strut classes, and a 99.2% and 97.8% rate for lumen and “other” structures, respectively.

As a secondary confirmation, comparisons were made in area measurements with the proprietary software (LightLab Imaging Inc., Westford, Massachusetts). Manual tracings of stent and lumen were made by a trained operator in three stented vessels in a different animal than that was used to train the automated classifier. Lumen area was calculated as the integrand of the radial location of the lumen boundaries in the Cartesian image while the stent area was calculated from a circle fitted to the detected stent struts within each frame (frames with less than one detected strut per quadrant were discarded). Differences in area measurements between the manual and automated methods are presented in the text as mean ± standard deviation, along with linear regression analysis and their respective Pearson correlation coefficients.

## Enhanced reconstruction method: Vessel Centerline Reconstruction

### Method Development

3D spatial reconstruction of OCT stent and lumen features required frames to be transformed about the path over which the images were acquired. Given uncertainty in catheter movement during imaging[[5](#_ENREF_5)], we used an established method that approximates the OCT imaging path as the vessel centerline[[6](#_ENREF_6)] reconstructed from isocentric planar angiography obtained at end-diastole[[7](#_ENREF_7), [8](#_ENREF_8)]. Briefly, two angiographic cines with defined rotational angles relative to the normal angiographic C-arm position and magnification factor were taken over several cardiac cycles. We ensured image isocenters were coincident. End-diastolic frames were manually selected (Supplemental Figure B.A) and thresholded to obtain binary images of the lumen (Supplemental Figure B.B), that was subsequently morphologically thinned[[9](#_ENREF_9)] to create candidates of centerline projections in each image (Supplemental Figure B.C). Corresponding points on projected centerlines in each planar image were identified based on a minimum distance threshold to the epipolar line (Supplemental Figure B.D).[[7](#_ENREF_7)] The 3D centerline was reconstructed from these paired points using stereoscopic relations based on rotational position and magnification of the angiographic system (Supplemental Figure B.E).[[7](#_ENREF_7)] A cubic spline function was used to smooth reconstructed points.

### Validation

In contrast to hybrid approaches that fuse angiography with intravascular ultrasound (IVUS), lack of EKG-gating with OCT limits the ability to temporally co-register frames and negate motion artifacts. Thus, registering all OCT frames to a dynamically changing but statically-approximated luminal centerline (i.e. at a single end-diastolic time point) may distort reconstructed features. As introduced uncertainties could have a significant impact when attempting to accurately reconstruct stents *in situ*, we attempted to address some of these concerns by examining first the accuracy in vessel centerline estimation from a single time point, and then quantifying deformation of stented-vessel centerline during different points of the cardiac cycle.

We tested the method of vessel centerline extraction in a phantom bifurcation model (Supplemental Figure B.F), considering a 20 to 110 degree range between paired angiographic frames. Reconstructed 3D path geometries (Supplemental Figure B.G) had an error in length of 1.7 ± 1.2 mm and in bifurcation angle of 8.4 ± 5.6 degrees (n = 14). Since the mathematical formulation of this reconstruction algorithm has previously been verified[[8](#_ENREF_8)], this error most likely indicates some discrepancy due to limited angiographic resolution (~3 pixels/mm).

We then tested sensitivity of the reconstructed centerlines to dynamic conditions found within *in vivo* settings by implanting stents in swine coronary arteries, imaging and comparing reconstructed geometries by measuring misalignment of downstream centerline landmarks on side and main branches, respectively. The vessel centerline of the stented segment did not deform considerably within the cycle (between diastolic and systolic phases, curvature κ = 0.13 ± 0.09, Δκ = 0.09 ± 0.09, n = 4 vessels, 6.0 ± 2.0 time points) nor between the cycles (curvature κ = 0.13 ± 0.09, n = 3 vessels). While the stented centerline remained relatively intact in length during pullback, the vessel rotated ~14 ± 15 degrees about its centerline during the cardiac cycle (n = 4 vessels, 6.0 ± 2.0 time points), thus contributing to uncertainty in the estimates of the position of the first frame on the centerline ~1.5 ± 1.0mm. Despite uncertainty in absolute stent position, reconstructed stent length varied by not more than 0.17 ± 0.12 mm – a value small and not significantly larger than the longitudinal resolution of the OCT itself (0.2 mm). This is most likely because stent imposes rigidity on the vessel, helping prevent excessive errors due to vascular deformation. Generated centerlines exhibited good reproducibility through various paired angiographic imaging angles (RAO/LAO; error of ~ 0.17 ± 0.10 mm in stent length, n = 4 vessels @ 12 angles). When combined, these *in vivo* errors were no larger than those in the isolated static phantom model and we concluded that dynamic forces and imaging angles do not contribute significantly to errors, beyond that already introduced by the low resolution of angiography.

## Enhanced reconstruction method: Point Set Registration and Surface Reconstruction

### Method Development

Fusion of the vessel centerline with OCT measurements provided a point cloud defining the stent struts in 3D space. An enhanced reconstruction of the stent was generated by registering an idealized stent design to the stent strut point cloud. The idealized stent skeleton was defined a priori (from images of an unrolled stent deployed *in vitro*) as the point set *Y* connected by NURBS curves $Q_{k}$, where *k* represents each continuous closed cell or open connector in the stent design (see definitions; Supplemental Figure D.b). As there is no one-to-one correspondence between stent skeleton, *Y*, and point set P of strut centroids obtained from transformed OCT frames, we matched the two point using a probability density estimation scheme. The ideal point set, Y, was represented as Gaussian centroids fit onto the OCT data points, P, using the method developed by Myronenko and Song.[[11](#_ENREF_11)] In this method, Gaussian centroids of the points are forced to move coherently as a group (Supplemental Figure C).

For each curve $Q_{k}$with trajectory *v*, we swept a section curve $C\left( u \right)$ representing the cross section of the stent (Supplemental Figure D.b), to create a NURBS surface representation of the surface $S_{k}$. Mathematically this is defined as:

$$S_{k} (u,v)=Q_{k} (v) + M(v)C(u) (2)$$

Where $M(v)$ is a 3 x 3 matrix representing the orientation of the section curve $C\left( u \right)$ at its new location. In general, a common solution to $M(v)$ is the Frenet relations.[[12](#_ENREF_12)] However, Equation 2 can generate incorrect surface approximations due to self-intersections and discontinuities[[13](#_ENREF_13)]. We therefore approximated $M\left( v \right)$first from the Frenet relations, and then iteratively rotated the local coordinate system of C on the trajectory to minimize rotation of between successive section curves ($S_{k}$ and $S_{k+1}$). A Boolean operation was then performed to unite all surfaces $S_{k}$ to form a watertight solid body defining the stent geometry that permitted computational simulation.

### Validation

The relative fit of the final, deformed stent shape model to actual OCT data was calculated by measuring displacement error between landmarks. Landmarks on the reconstructed surface were determined first finding the intersecting contour between stent structure and the OCT frame yielded contours of struts. These landmarks were then mapped to the detected OCT stent strut point cloud on 20 frames distributed equally along the length of the vessel (Supplemental Figure 4) The average displacement vector between landmarks was 16.6 μm (displacements not statistically significant; p = 0.67, n = 20 plane, 136 detected strut points, Supplemental Figure E). At a more regional level, there was also high correlation in both the cross sectional stent radius and lateral stent curvature for the OCT-derived parameters versus and our enhanced reconstructed geometry ($R_{Radius}^{2}=0.91, R_{curvature}^{2}=0.90 ;$ See manuscript Fig.4D). This demonstrates stent cross-sectional dimensions and stent shape are maintained during registration.

# Supplemental Figures


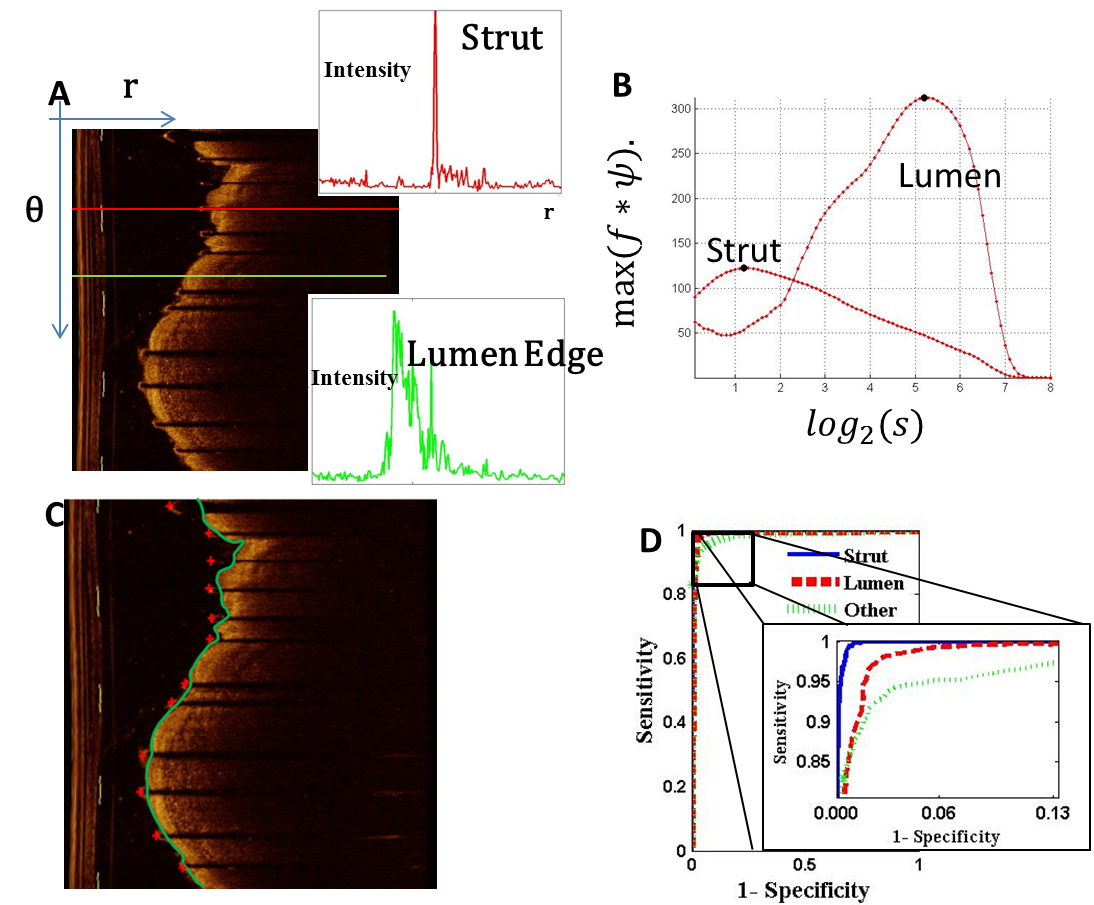


Supplemental Figure A: (A) Example of an optical coherence image in the polar coordinate system. A pixel stent strut candidate creates a trailing shadow in its A-line, when compared to a pixel candidate belonging to the lumen wall. (B) The convolution of each A-line signal with the negative normalized second-derivative of the Gaussian yields the LoG response across scales, and this coarse-to-fine tracking is called the scale space signature. Using features extracted from the scale space signature, one can classify structures as part of either (C) lumen (-) or stent strut (+). (D) Receiver operator characteristic curves illustrate the accuracy in classifying lumen and strut structures from the derived feature set and using a decision tree algorithm (Bootstrap aggregation).


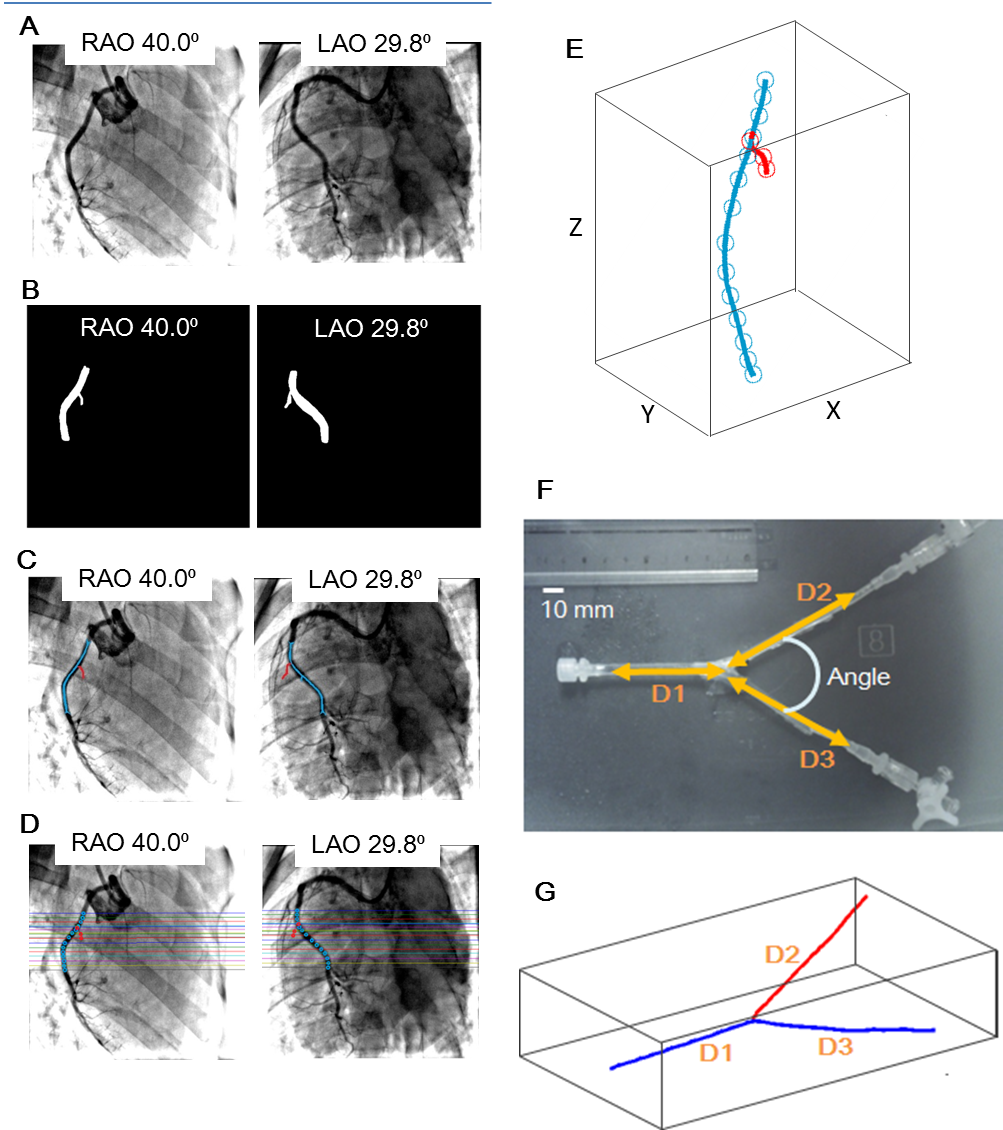


Supplemental Figure B: Method to reconstruct the centerline of the artery. From the original angiographic images taken from two different angles (A), binary images were generated by defining threshold and the Range of Interest (ROI). Then, the arterial edge was detected and thresholded to create a binary image of the lumen (B) that was subsequently morphologically thinned to create candidates of centerline projections in each image (C). After finding the points corresponding in both figures by epipolar constraints (D), the centerline was reconstructed (E). The method was validated first in the phantom model (F), of the coronary artery bifurcation. (G) Shows the reconstructed result of this phantom model. Three distances (D1-D3) and the angle were measured in the actual phantom model (F) and the reconstructed image (G) to analyze the static errors.


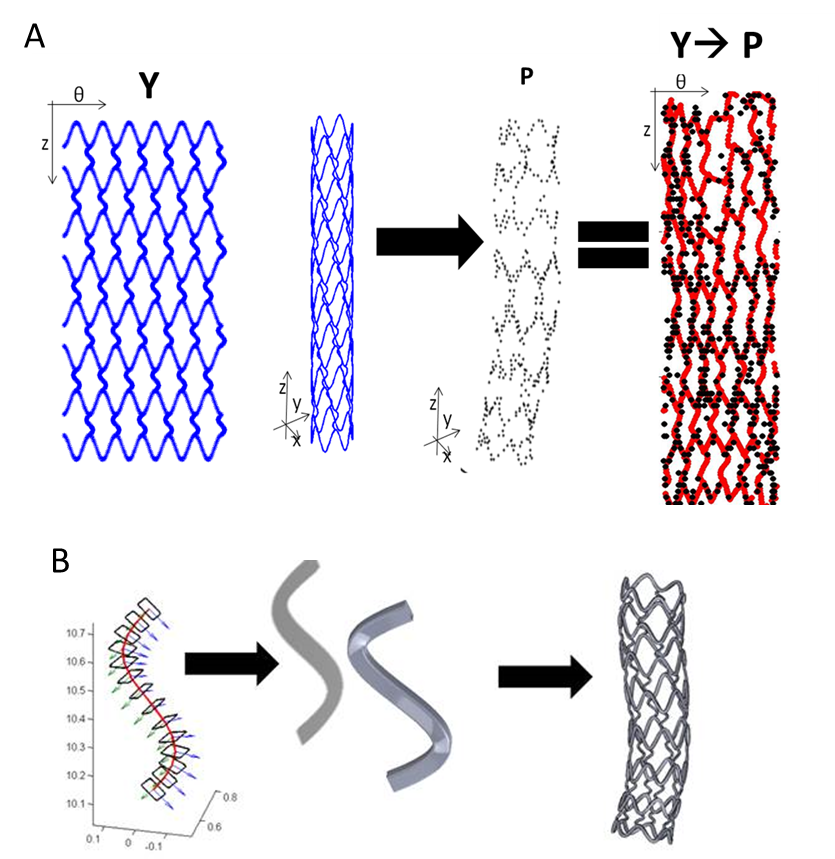


Supplemental Figure C: Schematic demonstrating the registration algorithm used to fit a data set Y representing the stent skeleton shape to a point set P of detected stent struts from OCT-angiography fusion. While this registration is performed in the Cartesian space (x-y-z), this is best visualized in the polar domain (θ-z plane). B. Surfaces were then built around the deformed skeleton using a swept cross-section to define the stent surface of each cell or connector, and then Boolean operations were performed to unite all surface elements and define a single, solid watertight body representing the stent.


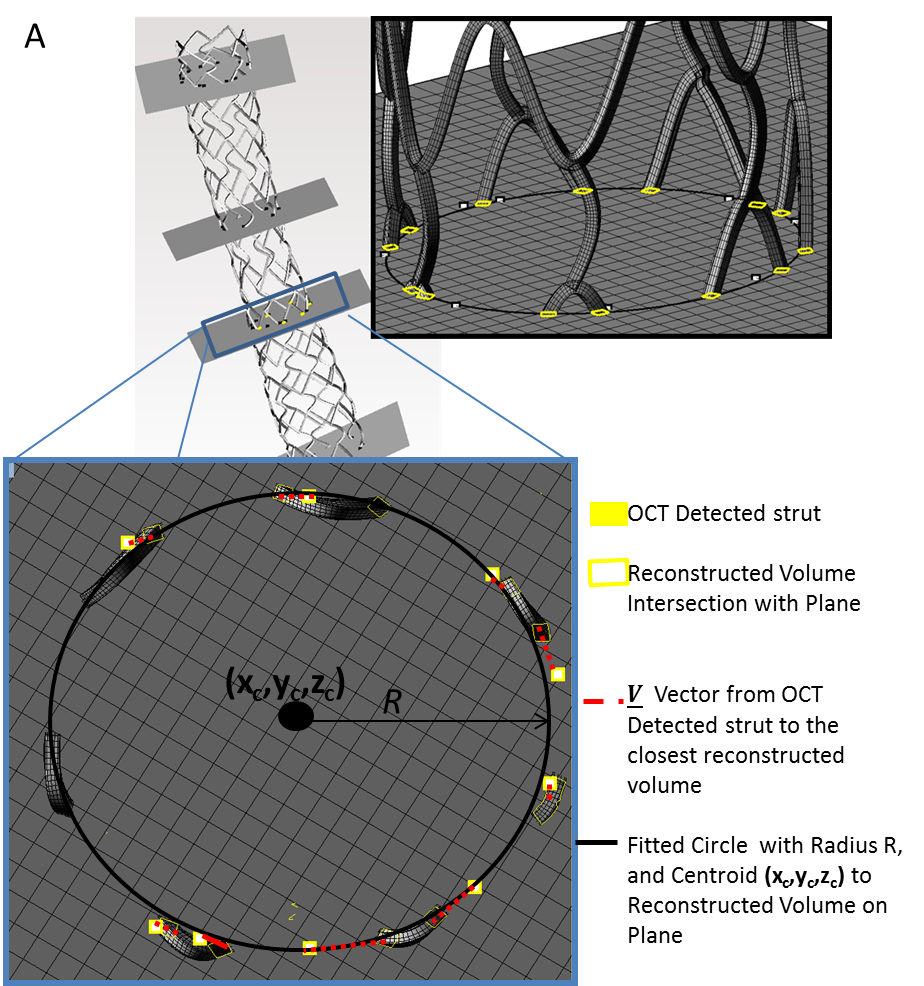


Supplemental Figure D: Reconstructed stent surface geometry shown for a single data set of a sub-optimally deployed closed-cell stent. Landmarks were defined by mapping each OCT-detected strut (White marker) on its respective OCT frame and the closest surface structure on that frame (yellow). The displacement between each of these landmarks is defined by the difference vector V. Total error in fit was calculated by averaging difference vectors across all landmarks, defined as “average strut displacement error”. The p-value for testing the significance of vector differences between OCT and reconstruction landmarks was determined via the multivariate Hotelling's T^2^ statistic, with separate vector components representing the multiple variables. Stent area was calculated as the circle fit to the centroids of the contours (in black).


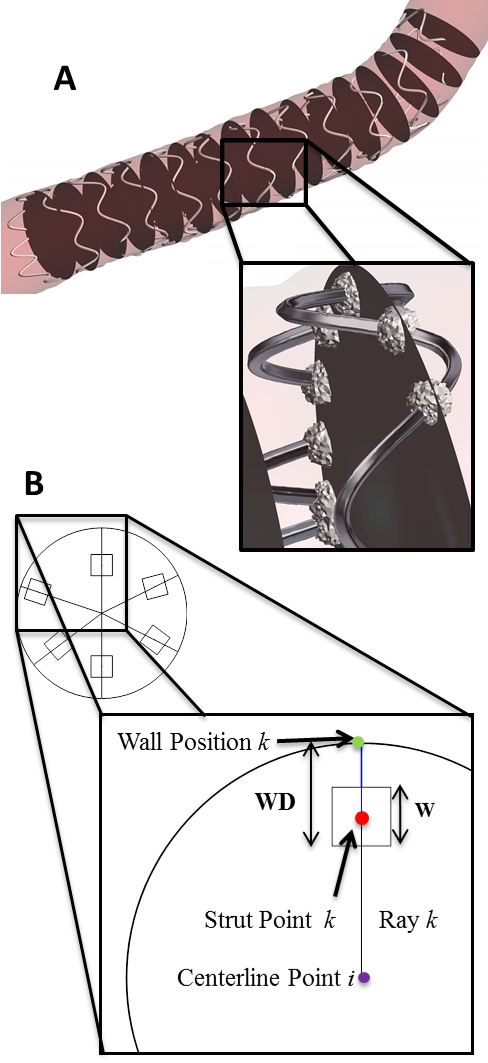


Supplemental Figure E: Computational simulation data was post-processed by extracting point-wise measurements of strut points defined as (A) the intersection of the stent body and 20 equidistant planes perpendicular to- and equidistant along- the vessel centerline. Also shown is the spherical volume taken around each detected strut intersection with the plane. (B) Strut points (in red) were defined by the intersection of the planes and the stent body. The corresponding wall position of each strut point was defined by the intersection of the vessel wall boundary and a ray (in black) extending from the centerline point on the plane (in purple) through the strut point k to the wall, shown in green. The distance separating the strut point and the wall position was defined as the wall distance.

# Supplemental References

1. Bezerra HG, Costa MA, Guagliumi G, Rollins AM, Simon DI. Intracoronary optical coherence tomography: a comprehensive review clinical and research applications. JACC Cardiovasc Interv. 2009;2(11):1035-46.

2. Tsantis S, Kagadis GC, Katsanos K, Karnabatidis D, Bourantas G, Nikiforidis GC. Automatic vessel lumen segmentation and stent strut detection in intravascular optical coherence tomography. Med Phys. 2012;39(1):503-13.

3. Zhang L, Bao P. Edge detection by scale multiplication in wavelet domain. Pattern Recognition Letters. 2002;23(14):1771-84.

4. Haralick RM, Shapiro LG. Computer and Robot Vision: Addison-Wesley Longman Publishing Co., Inc.; 1992. 630 p.

5. Tu S, Holm NR, Koning G, Huang Z, Reiber JH. Fusion of 3D QCA and IVUS/OCT. Int J Cardiovasc Imaging. 2011;27(2):197-207.

6. Wahle A, Prause PM, DeJong SC, Sonka M. Geometrically correct 3-D reconstruction of intravascular ultrasound images by fusion with biplane angiography--methods and validation. IEEE Trans Med Imaging. 1999;18(8):686-99.

7. Faugeras O, Luong Q-T, Papadopoulo To. The geometry of multiple images : the laws that govern the formation of multiple images of a scene and some of their applications. Cambridge, Mass.: MIT Press; 2001. xxiv, 644 p. p.

8. Shechter G, Shechter B, Resar JR, Beyar R. Prospective motion correction of X-ray images for coronary interventions. IEEE Trans Med Imaging. 2005;24(4):441-50.

9. Lam L, Lee SW, Suen CY. Thinning Methodologies-A Comprehensive Survey. IEEE Trans Pattern Anal Mach Intell. 1992;14(9):869-85.

10. Prause GP, DeJong SC, McKay CR, Sonka M. Towards a geometrically correct 3-D reconstruction of tortuous coronary arteries based on biplane angiography and intravascular ultrasound. Int J Cardiovasc Imaging. 1997;13(6):451-62.

11. Myronenko A, Song X. Point set registration: coherent point drift. IEEE T Pattern Anal. 2010;32(12):2262-75.

12. Klok F. Two moving coordinate frames for sweeping along a 3D trajectory. Computer Aided Geometry Design. 1986;3(3):217-29.

13. Piegl LA, Tiller W. The NURBS book. 2nd ed. ed. Berlin ; London: Springer; 1997.
